# Facile Access to Graphene Oxide from Ferro-Induced Oxidation

**DOI:** 10.1038/srep17071

**Published:** 2016-01-28

**Authors:** Chao Yu, Cai-Feng Wang, Su Chen

**Affiliations:** 1 State Key Laboratory of Materials-Oriented Chemical Engineering and College of Chemistry and Chemical Engineering, Nanjing University of Technology, Nanjing 210009, P.R. China.

## Abstract

Methods allowing the oxidation of graphite to graphene oxide (GO) are vital important for the production of graphene from GO. This oxidation reaction has mainly relied on strong acid strategy for 174 years, which circumvents issues associated with toxicity of reagent and product, complex post-treatment, high cost and waste generation. Here, we report a green route for performing this oxidization reaction via a ferro-induced strategy, with use of water, potassium ferrate (Fe(VI)) and hydrogen peroxide (H_2_O_2_) as reagents, to produce about 65% yield of GO (vs. 40% for Hummers’ method, the most commonly used concentrated acid strategy) and non-toxic by-products. Moreover, GO produced from this new method shows equivalent performance to those reported previously. This H_2_SO_4_-free strategy makes it possible to process graphite into GO in a safe, low-cost, time-saving, energy-efficient and eco-friendly pathway, opening a promising avenue for the large-scale production of GO and GO-based materials.

Since 2004, graphene and graphene oxide (GO) have sparked tremendous interest as an emerging field of interdisciplinary science across a broad spectrum of disciplines, involving chemistry, physics, materials science and nanoscience^1^,^2^,^3^,^4^,^5^,^6^. Currently, chemical or thermal reduction methods are the center routes for the large-scale production of graphene from GO precursor due to its versatility and easy processability^7^,^8^,^9^,^10^. After discovered by Schafhaeutl in 1840^11^, GO was synthesized by Brondie *et al.* with the use of potassium chlorate (KClO_3_) as the oxidant to oxidize the graphite in nitric acid in^12^. Then, Staudenmaier optimized this protocol in 1898, by adding the chlorate in multiple aliquots over the reaction course to minimize the risk of explosion and using concentrated sulfuric acid (H_2_SO_4_) for the enhancement of acidity in the mixture^13^. Nearly 10 years later, Charpy developed this method by using the potassium permanganate (KMnO_4_) as the oxidant to oxidize the graphite in H_2_SO_4_ below 45 °C^14^. The same procedure was then optimized and scaled up in 1958, which is called Hummers’ method and extensively used today^15^. Hummers treated the raw graphite with KMnO_4_ and NaNO_3_ in concentrated H_2_SO_4_ and applied this procedure to achieve a multi-grams scale. Although modified versions have also been proposed by the researchers^16^,^17^,^18^, little has been altered. By now, this dominating oxidation protocol still highly depends on the abundant strong acid, which often involves environmental contamination, expensive cost, consuming time during treatment and purification process, limiting its industrial commercialization. The breakthrough in green synthesis of GO is therefore highly expected.

Here, we report here an alternative, facile and green method for the production of GO, referred to as ferro-induced production of GO (FIGO), by only using water, potassium ferrate (Fe(VI)), hydrogen peroxide (H_2_O_2_), and graphite as starting materials. In contrast to previous methods, this FIGO approach offers the following advantages: (1) the combination of Fe(VI) aqueous solution and H_2_O_2_ represents an ideal substitute for KMnO_4_ and strongly corrosive acids. Peng *et al.* reported an green approach to GO production using the Fe(VI) and H_2_SO_4_^19^. However, FIGO is an unprecedented way to produce GO in the absence of H_2_SO_4_; (2) the FIGO protocol with merits of simple post-treatment, high yield, time-saving process, low cost and relatively green chemistry, makes it compelling for large-scale preparing of GO in industry; (3) the as-prepared hydrophilic GO presents an overall extent of oxidation similar to previously reported GO, but does easy-to-perform. Since 1840, GO synthesis has gone through 175 years of acid-based history. Yet, these advances are encouraging, and FIGO might pave a reliable path to change current situation.

## Results

**Figure 1:**
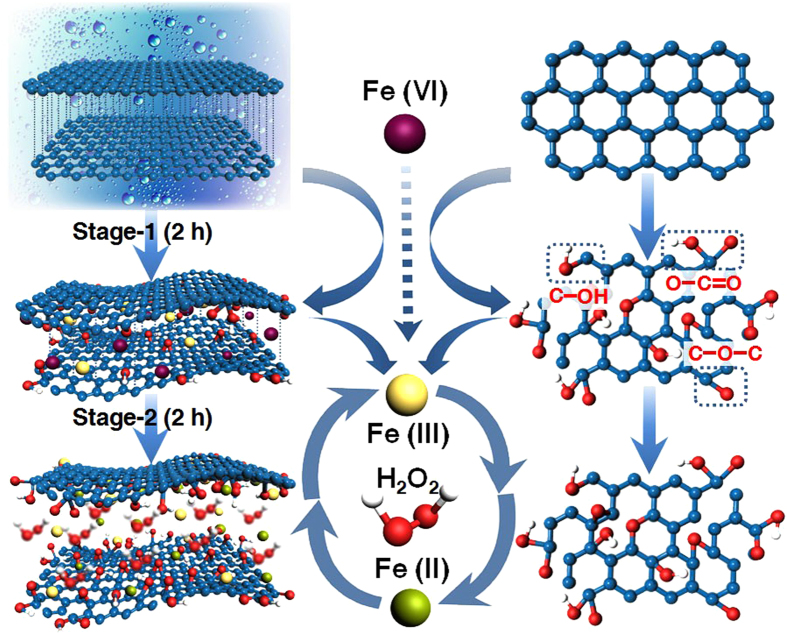
Schematic representation of FIGO procedure from initial raw graphite to targeted GO. The raw graphite is partially oxidized by Fe(VI) at **Stage-1**. This process rearranges the ordered structure of carbon atoms that oxygen atoms begin to enter and enlarge the graphitic spacing. In **Stage-2**, H_2_O_2_ react with Fe(III) to reoxidize the graphite. The two oxidation processes turn the raw graphite into GO.

The present FIGO approach is simple and quite general, involving two-stage synthesis (Fig. 1) and purification processes. Initially, we added 8 wt equiv of Fe(VI) aqueous solution (148 g/L) to oxidize 1 wt equiv of graphite for 2 h treatment at 50 °C (**Stage-1)**. After introducing hydrogen peroxide (H_2_O_2_) into reaction solution under ultrasonic environment, the secondly oxidative cutting took place for another 2 h (**Stage-2**). Finally, we carried out 2 h purification to afford 65% yield of GO (Supplementary Fig. S1). In contrast to Hummers’ method, which produces *ca.* 40% yield of GO from the treatment of graphite with KMnO_4_ and NaNO_3_ in concentrated H_2_SO_4_ for 33 h of complex synthesis and purification processes, this new FIGO strategy using Fe(VI) and H_2_O_2_ as oxidants is much more simple and fast, as well as higher production yield. (Supplementary Fig. S1).

As shown in Fig. 1, this wet transfer method for oxidation of graphite highly replies on a diffusive-controlled process, where the oxidant is needed to intercalate into graphitic interlayer through liquid transportation^20^. Carbon atoms of graphite in a single two-dimensional (2D) layer are arranged in a honeycomb-like lattice, and each layer is held together because of weak van der Waals. In **Stage-1**, powerful Fe(VI) oxidant by aid of water may attack the electron-rich moieties of localized defects in graphite sheet to enable a non-toxic decomposition^21^,^22^. During the propagation within layers, the introduction of deionized water rather than strong acids with higher viscosity allows Fe(VI) well-dispersed in the interlayer. Analytical results from UV/vis absorption, optical microphotographs, FT-IR spectra, Raman spectra, and X-ray photoelectron spectroscopy (XPS) demonstrate that the combination of Fe(VI)/H_2_O leads to the partial unzipping of graphitic layers, as well as the partial oxidation of graphite and the formation of C–O, and C = O bonds along the peripheral areas (Supplementary Fig. S3–S6). Meanwhile, XPS data suggest that Fe(III) species from the decomposition of Fe(VI) bound to the layer regions of the product (Supplementary Table S1)^23^. In fact, the breaks and stretches of C–C bonds in **Stage-1** may further weaken the neighboring bonds to allow them more vulnerable for the second attack. By feed of H_2_O_2_ (30 wt%) in **Stage-2**, this mixture turns into a typical Sono-Fenton system^24^,^25^, further enhancing oxidation reaction. In this system, the remaining Fe(III) and H_2_O_2_ serve as a catalyst and a radical producer, respectively, while the ultrasound serves as an accelerator and separator. This Sono-Fenton reaction allows the generation of a plenty of (**·**OH and **·**OH_2_) radicals, along with the formation of an effective cycle between Fe(III) and Fe(II). On the basis of partially oxidized graphite via Fe(VI) oxygenation and quite low viscosity of the whole system, the Fenton solution has an easy access to infiltrate into the interlayers of graphite, guaranteeing the occurrence of the recycle redox reaction in the interlayer and decorating both sides of the flake sheet. Meanwhile, ultrasound wave creates acoustic cavitation, facilitating the exfoliation of graphite oxide to GO. Thus, FIGO is an unfolding oxidation process progressing from edge to inner areas of layered sheets to give higher yield of GO.

**Figure 2:**
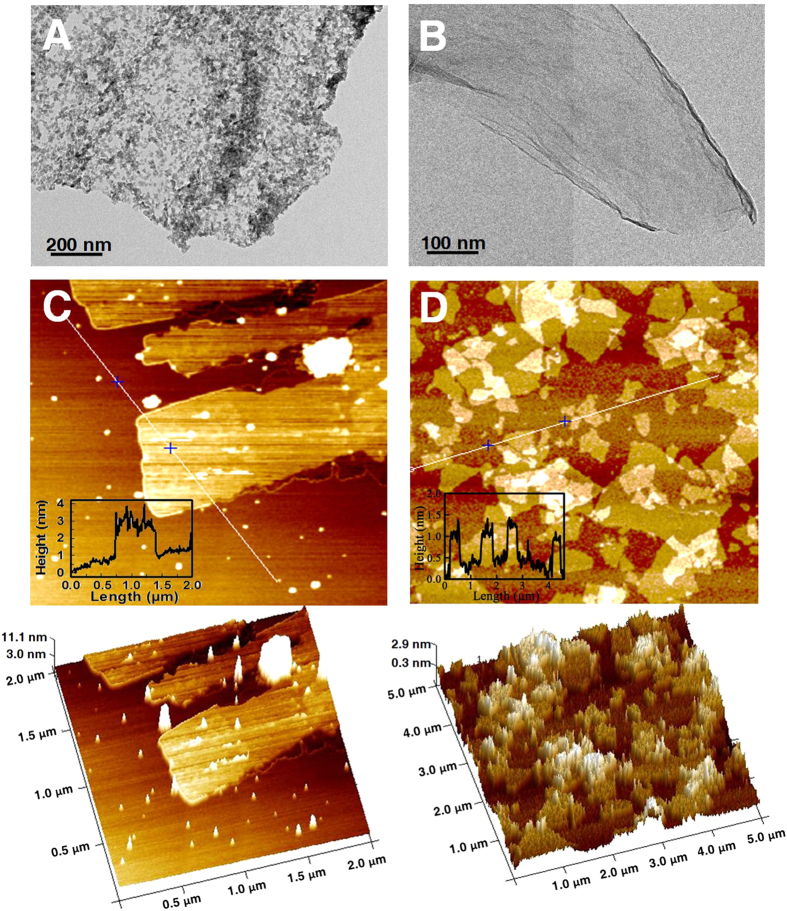
The resultant GO before and after purification. (**A**,**B**) TEM observation of as-prepared GO without purification (sample 1, (**A**)) and after purification (sample 2, (**B**)). (**C**,**D**) AFM images and corresponding height profiles of the surface of sample 1 (**C)** and sample 2 (**D**). The 3D contour images are shown below.

Based on these two-stage synthesis process, a brown aqueous solution of as-prepared GO products (sample 1) was obtained, which turns to light yellow after purification, referred to as sample 2. Figure 2 shows the AFM and TEM images of samples 1 and 2. It seems that some graphite particles distribute around the interior and edges of the GO platelets in sample 1 (Fig. 2A,C), whereas after purification pure GO product with platelet morphology is achieved (Fig. 2B, Fig. S8). AFM further indicates the GO flake of sample 2 has thickness of ~1.1 nm (Fig. 2D), which is approximate to one-atom-thick GO^26^.

**Figure 3:**
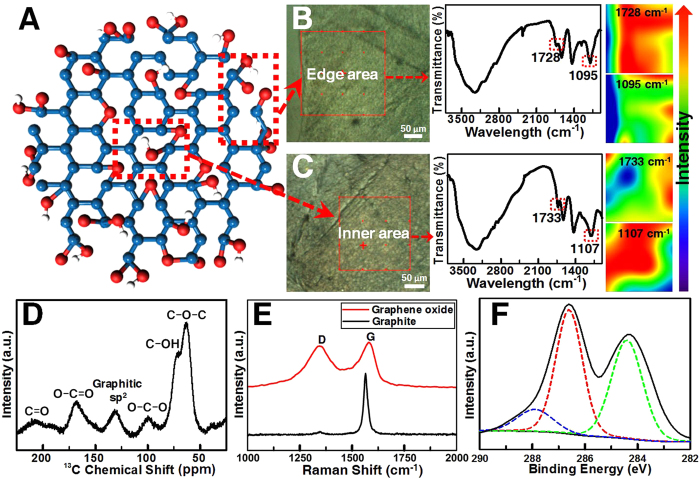
Characterization of GO prepared by FIGO. (**A**)Schematic representation of structure of GO. (**B,C**) Optical images (left), IR spectra (middle), and IR images (right) of a GO flake. B: edge area; C: inner area. (**D**) Solid-state ^13^C MAS NMR spectrum (100.62 MHz) of GO. (**E**) Raman spectra of graphite and GO. (**F**) XPS “C 1 s” peak of GO.

To investigate the “oxo” functionalities of GO prepared from FIGO, we conducted various spectroscopic characterizations (Fig. 3). Figure 3B,C, and Supplementary Fig. S10–13 depict the IR spectra and IR images of edge areas and inner areas of GO flakes. There are characteristic functional groups in both areas of GO: strong and broad absorption bands at 3320–3480 cm^−1^ originating from O-H stretching vibrations (carboxyl and hydroxyl groups); bands at 1637 cm^−1^ assigned to unoxidized C=C graphitic domains; bands at 1720–1740 cm^−1^ associated with C=O stretching vibration; bands around 1400–1450 cm^−1^ corresponding to O-H bending vibration; the intense peaks at 1060–1140 cm^−1^ attributed to the C-O alkoxy group. Since no distinctive peak could be found in raw graphite (Supplementary Fig. S9), the observation of oxygen-containing functional groups in IR spectrum reveals that certain fraction of “oxo” functionalities were built on the backbone of carbon. Moreover, IR images exhibit chemical changes from the edge area to inner area of GO flakes, and directly illustrate the distribution of the oxygen-containing species through color variation that red color means high intensity and blue color represents low intensity (Fig. 3B,C; Supplementary Fig. S10–13). The results show that carboxylic groups (~1730 cm^−1^) are much richer on the edge area while epoxy (~1100 cm^−1^) and hydroxyl (~1430 cm^−1^) groups are more abundant in the inner area of GO flakes, which are consistent with the Lerf-Klinowski model that epoxy groups together with hydroxyl trap into the in-plane aromatic rings, while carboxyl groups most likely attach to the edges of the GO platelet^27^.

We also performed ^13^C magic-angle spinning (MAS) nuclear magnetic resonance (NMR) experiments on FIGO-prepared GO sample, which displays six broad resonances (Fig. 3D). The signal at 63 ppm is unambiguously from epoxidation and the shoulder at 70 ppm is attributed to hydroxylated carbons^28^. The peak at 100 ppm should be assigned to the carbons of five- and six-membered-ring lactols, while graphitic sp^2^ carbon resonates at 131 ppm. The carbonyl groups are responsible for the resonances appearing at 167 and 205 ppm. The results imply that graphite was subject to a high level of oxidation in the FIGO process.

Consistent with IR and ^13^C NMR spectra, Raman spectra confirm the oxygen functionalities of FIGO-prepared GO (Fig. 3E). The G peak at 1580 cm^−1^ is due to the high-frequency *E*_2g_  phonon at **Γ** and the D peak at 1340 cm^−1^ corresponds to the *A*_1g_ breathing modes at **K**^29^. The prominent D peak in the spectrum of GO can be correlated to the defect density introduced during the FIGO process. Note that when sp^3^ defects are embedded into the basal plane, a decrease of the sp^2^ domains, a sharp increase in D/G intensity ratio, and broadening of all the bands are observed in comparison with that of graphite. Those Raman fingerprints are typical for GO.

XPS provides further information about the elemental composition and chemical environment of carbon atoms in GO. The C/O ratio of 2.2 indicates the successful introduction of oxygen atoms into the ordered arrangement of graphite structure (Supplementary Fig. S14). The C 1 s XPS spectra show that the non-oxygenated ring carbon (C−C/C=C) is located at 284.3 eV, while 286.6 and 287.8 eV correspond to the carbon of alcohols and epoxides (C-OH/C-O-C) and carbon from carboxyl groups (C=O/O−C=O), respectively (Fig. 3F). These above results obtained from IR, Raman, ^13^C NMR, and XPS spectra clarify the “oxo” functionalities and chemical composition of GO prepared by FIGO, which display no anomalous behavior in comparison with those of other work^30^, confirming the successful synthesis of GO via FIGO strategy.

**Figure 4:**
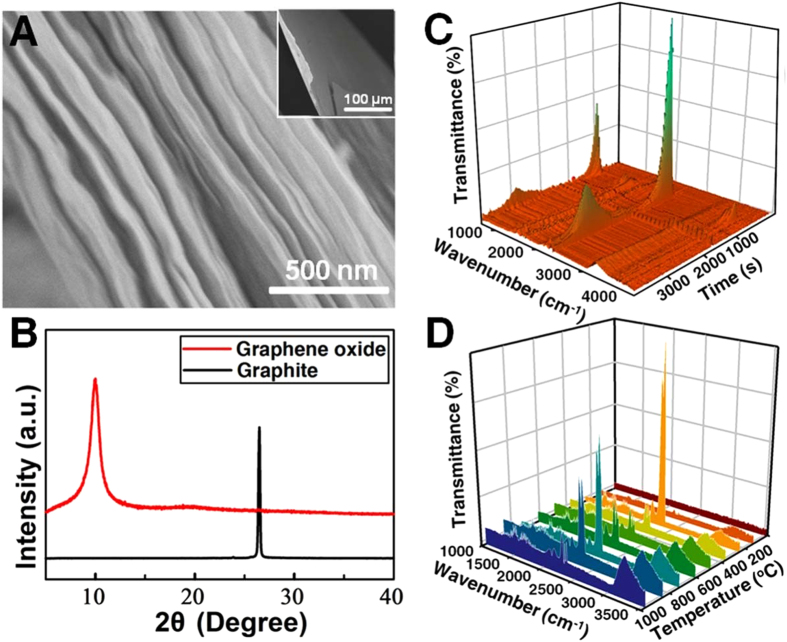
Morphology and characterization of FIGO-prepared GO paper. (**A**) SEM side-view image of folded ~1-μm-thick GO paper. (**B**) XRD pattern of GO paper. (**C**) 3D IR profile (30 to 1000 °C) and (**D**) FT-IR spectra of evolved gases produced from GO combustion under N_2_ atmosphere.

Finally, vacuum filtration of aqueous dispersions of GO yielded the assembly of free-standing paper-like GO materials. The fracture edge of a GO paper was imaged via scanning electron microscopy (SEM). As shown in Fig. 4A, GO nanosheets are well layered in a nearly parallel fashion with some degree of wrinkle and waviness. The interlayer distance was determined via X-Ray Diffraction (XRD) analysis (Fig. 4B). The raw graphite gives detectable peak at 2θ = 26.5°, corresponding to the interlayer distance of 3.3 Å. Whereas, in the XRD pattern of the GO paper, the graphitic peak almost disappears and the peak around 10° becomes more pronounced and broader, through which the interlayer distance was calculated to be 8.8 Å. The increase of interlayer distance for GO paper might be ascribed to the lattice distortion that occurs in the controlled oxidative scission, as well as the formation water monolayers occupied between the GO nanosheets^31^. Moreover, thermogravimetric analysis (TGA)/FT-IR spectra were performed to record the pyrolysis procedure of the GO paper. Slight mass loss occurred upon heating above 75 °C, presumably owing to the evaporation of loosely bounded or adsorbed water and gas molecules, and a major mass loss was observed at 200 °C (Supplementary Fig. S15). 3D FT-IR profile shows the appearance of CO_2_ and H_2_O (2348 cm^−1^ and 3225 cm^−1^) gas at 160 °C, and their contents gradually elevate with the heating temperature to reach the highest at 200 °C (Fig. 4C,D). A much slower mass loss was observed between 500 and 1000 °C, which should be associated with the decomposition of more stable O-species. The combustion of GO into grapheme quantum dots is also available (Supplementary Fig. S16–20).

## Discussion

We have demonstrated herein a new simple and green method for production of GO with high yield of 65% in 6 hours, by using Fe(VI) and H_2_O_2_ as reagents to oxidize the raw graphite in water. This ferro-induced strategy is much simpler and superior over the Hummers’ method which produces *ca.* 40% yield of GO from the treatment of graphite with KMnO_4_ and NaNO_3_ in concentrated H_2_SO_4_ after 33 h of complex synthesis and purification processes. Furthermore, free-standing GO paper has been obtained with interlayer distance of 8.8 Å. These studies contribute an alternative safe, inexpensive, time-saving, high-yield and eco-friendly route over traditional synthetic methods for preparation of GO, which are valuable for large-scale production and application of GO and its relative materials.

## Methods

### Materials

Graphite (120 mesh) and hydrogen peroxide (H_2_O_2_) (AR, 30 wt% in water) were received from Aladdin Industrial Corporation and used without any further purification. Potassium ferrate (Fe(VI)) of high purity was prepared according to the literature method^32^ and directly used after washing more than 4 times.

### Ferro-induced production of GO

In a typical example of ferro-induced production of GO (FIGO), we added graphite flakes (1.0 g, 1 wt equiv) into a mixture of deionized water (54 ml) and Fe(VI) (8.0 g, 8 wt equiv) in a reaction vessel initially. Then the vessel was immersed in a thermostatic oil bath at 50 °C under stirring with stirring speed of 250 rpm for 2 h. After the system was cooled to ambient temperature, H_2_O_2_ (100 ml) was added, and the mixture was allowed to stir under the ultrasonic treatment (400 W) and pH = 3 for another 2 h. Subsequently, the product was centrifuged (8000 rpm) in succession with 30% HCl (3×) and ethanol. For each wash, the mixture was washed for 20 mins and the supernatant was decanted away. The mixture was then washed with water to remove the remaining pristine graphite. Finally the aqueous GO solution was dried on the funnel under a continuous air flow.

### Preparation of GO paper

GO paper was made by vacuum filtration of the as-purified GO dispersion (0.08 mg/ml) through a mixed cellulose ester filter membrane (47 mm in diameter, 0.2 μm pore size), followed by air drying and peeling from the filter. Samples of GO paper prepared in this manner were cut by a razor blade into approximate size for testing without further modification.

### Measurements

#### Transmission electron microscopy (TEM) and scanning electron microscopy (SEM)

The surface microstructure of FIGO-obtained GO were examined with a JEOL JEM-2100 TEM. A drop of GO solution was placed on a lacey carbon film that was left to dry before being transferred into the TEM sample chamber. The morphology of the cross section of the resultant GO paper was investigated by SEM with a QUANTA 200 (Philips-FEI, Holland) at 30.0 kV. GO paper used for SEM measurement were cut to expose their inner structure.

#### Atomic force microscopy (AFM)

GO for AFM were prepared by drop-casting the suspensions onto the freshly cleaved mica. Imaging was accomplished under ambient conditions with Bruker Dimension Icon scanning probe microscope in the tapping mode of operation.

#### Infrared imaging (IR imaging)

IR images of the GO paper were performed on a Thermo Scientific Nicolet iN10 infrared microscope equipped with a liquid nitrogen cooled MCT detector (Thermo Electron Corporation, USA). IR microscopy data were collected using reflection mode. IR spectra were captured using an aperture size of 50 μm by 50 μm and were recorded over a range of 650–4000 cm^−1^. An analysis of the IR microscopy data was performed using OMNIC picta software (Thermo Electron Corporation, USA).

#### Nuclear magnetic resonance (NMR) measurement

Solid state ^13^C magic-angle spinning (MAS) NMR spectra were obtained on a Bruker Avance 400 D instrument operating in a 9.4 T magnetic field (^13^C, 100.6 MHz) using a 4 mm diameter solid-state probe head at 15.0 kHz without decoupling. ZrO_2_ rotors were used with an approximately 90.0 mg amount of GO samples rotating at a 10.0 kHz speed. Tetramethylsilane was used as the external reference material (δ = 0 ppm for ^13^C).

#### Raman measurement

Raman spectra were recorded by the single scan generated by the Horiba HR 800 Raman system equipped with a 514.5 nm laser.

#### X-Ray Diffraction (XRD) measurement

The XRD patterns were recorded on a Bruker D8 Advance X-ray diffractometer (40 kv, 25 mA, Cu Kα radiation, λ = 1.5418 Å) at room temperature. The data was collected in the range of 5° < 2θ < 60 ° with the scan rate of 2 ° min^−1^ and step width of 0.02 °.

#### X-ray photoelectron spectroscopy (XPS) measurement

XPS spectra of the GO were collected on an ES-CAIAB250 XPS system with Al/K α as the source, and the energy step size was set as 0.100 eV.

#### Thermal stability measurement

The thermal stability experiments were performed using a thermogravimetric analysis (Model TGA92, Setaram, France.) and a IR spectrometer (Vector 22 type, Bruker, Germany) equipped with an IR gas cell. The samples were combusted in N_2_ at the temperature ranging from 30 to 1000 °C (20 °C/min). Three dimensional (3D) IR profile was done with OPUS 6.5 software.

## Additional Information

**How to cite this article**: Yu, C. *et al.* Facile Access to Graphene Oxide from Ferro-Induced Oxidation. *Sci. Rep.* **6**, 17071; doi: 10.1038/srep17071 (2016).

## Supplementary Material

Supplementary Information
