# Supplementary material for: Facile Access to Graphene Oxide from Ferro-Induced Oxidation
Source: Sci Rep. 2016 Jan 28;6:17071. doi: 10.1038/srep17071 (PMC4730196; doi:10.1038/srep17071)
Supplement: Supplementary Information [file srep17071-s1.doc]

**Supplementary Information**

**Facile Access to Graphene Oxide from Ferro-Induced Oxidation**

*Chao Yu, Cai-Feng Wang, and Su Chen**

State Key Laboratory of Material-Oriented Chemical Engineering and College of Chemistry and Chemical Engineering, Nanjing University of Technology, Nanjing 210009 (P. R. China)

**Table of contents**

1. **Characterization and Results**
2. **Figure S1.** Comparison between Hummers’ method and FIGO for preparation of GO.
3. **Figure S2.** UV/vis absorption spectrum of Fe(VI) in reaction solution as a function of time (0~120 mins).
4. **Figure S3.** Optical microphotographs of partially oxidized graphite after the Stage-1 of FIGO and corresponding FT-IR spectra from certain areas.
5. **Figure S4.** Raman spectrum of partially oxidized graphite obtained in **Stage-1**.
6. **Figure S5.** XPS curve of partially oxidized graphite obtained in **Stage-1**.
7. **Figure S6.** C 1s curve of partially oxidized graphite obtained in **Stage-1**.
8. **Figure S7.** Photographs of the as-prepared GO without purification (sample 1) and after purification (sample 2).
9. **Figure S8.** TEM image of sample 2.
10. **Figure S9.** FTIR spectrum of graphite. There are no distinctive peaks in raw material.
11. **Figure S10.** Optical and IR images of GO flake prepared via FIGO at edge area.
12. **Figure S11.** Optical and IR images of GO flake prepared via FIGO at edge area.
13. **Figure S12.** Optical and IR images of GO flake prepared via FIGO at inner area.
14. **Figure S13.** Optical and IR images of GO flake prepared via FIGO at inner area.
15. **Figure S14.** XPS curve of partially oxidized graphite.
16. **Figure S15.** TG and DTG curves of GO prepared by FIGO.
17. **Figure S16.** Photographs of the FIGO-obtained GO and GQDs under daylight and UV light (λ_ex_=365 nm).
18. **Figure S17.** PL spectra of GQDs aqueous solution at different excitation wavelength (in 10 nm increment starting from 310 nm to 400 nm).
19. **Figure S18.** UV-vis absorption spectrum of GQDs aqueous solution.
20. **Figure S19.** Time-resolved fluorescence decay curves of GQDs measured at (405 nm laser excitation.
21. **Figure S20.** Photograph of the fluorescent pattern by screen printing and inkjet printing under UV light.
22. **Figure S21.** Raman spectrum of grapheme prepared from FIGO-prepared GO.
23. **Figure S22.** Optical image and FTIR spectrum of grapheme prepared from FIGO-prepared GO.
24. **Figure S23.** SEM micrograph of FIGO-prepared GO.
25. **Table S1.** XPS determined compositions of samples from Stage-1 and Stage-2.

**Characterization**

*Transmission electron microscopy (TEM) and scanning electron microscopy (SEM):* The surface microstructure of FIGO-obtained GO were examined with a JEOL JEM-2100 TEM. A drop of GO solution was placed on a lacey carbon film that was left to dry before being transferred into the TEM sample chamber. The morphology of the cross section of the resultant GO paper was investigated by SEM with a QUANTA 200 (Philips-FEI, Holland) at 30.0 kV. GO paper used for SEM measurement were cut to expose their inner structure.

*Atomic force microscopy (AFM):* GO for AFM were prepared by drop-casting the suspensions onto the freshly cleaved mica. Imaging was accomplished under ambient conditions with Bruker Dimension Icon scanning probe microscope in the tapping mode of operation.

*Infrared imaging (IR imaging):* IR images of the GO paper were performed on a Thermo Scientific Nicolet iN10 infrared microscope equipped with a liquid nitrogen cooled MCT detector (Thermo Electron Corporation, USA). IR microscopy data were collected using reflection mode. IR spectra were captured using an aperture size of 50 μm by 50 μm and were recorded over a range of 650-4000 cm^-1^. An analysis of the IR microscopy data was performed using OMNIC picta software (Thermo Electron Corporation, USA).

*Nuclear magnetic resonance (NMR) measurement*: Solid state ^13^C magic-angle spinning (MAS) NMR spectra were obtained on a Bruker Avance 400D instrument operating in a 9.4 T magnetic field (^13^C, 100.6 MHz) using a 4 mm diameter solid-state probe head at 15.0 kHz without decoupling. ZrO_2_ rotors were used with an approximately 90.0 mg amount of GO samples rotating at a 10.0 kHz speed. Tetramethylsilane was used as the external reference material (δ = 0 ppm for ^13^C).

*Raman measurement:* Raman spectra were recorded by the single scan generated by the Horiba HR 800 Raman system equipped with a 514.5 nm laser.

*X-Ray Diffraction (XRD) measurement:* The XRD patterns were recorded on a Bruker D8 Advance X-ray diffractometer (40 kv, 25 mA, Cu Kα radiation, λ = 1.5418 Ȧ) at room temperature. The data was collected in the range of 5° < 2θ < 60 ° with the scan rate of 2 ° min^-1^ and step width of 0.02 °.

*X-ray photoelectron spectroscopy (XPS)* measurement: XPS spectra of the GO were collected on an ES-CAIAB250 XPS system with Al/K α as the source, and the energy step size was set as 0.100 eV.

*Thermal stability measurement:* The thermal stability experiments were performed using a thermogravimetric analysis (Model TGA92, Setaram, France.) and a IR spectrometer (Vector 22 type, Bruker, Germany) equipped with an IR gas cell. The samples were combusted in N_2_ at the temperature ranging from 30 to 1000 ^o^C (20 ^o^C/min). Three dimensional (3D) IR profile was done with OPUS 6.5 software.

*Measurement of optical properties*: UV-vis absorption spectra were recorded by a UV-vis spectrometer (Lambda 950, Perkin-Elmer). Photoluminescence (PL) measurements were carried out on a Varian Cary Eclipse spectrophotometer. Fluorescence decay time was measured based on the Leica SP5 FLIM system using a 405 nm laser as the excitation source.

*Quantum yield (QY) calculation:* QY was measured according to the established procedure (J. R. Lakowicz, Principles of Fluorescence Spectroscopy, Kluwer Academic/Plenum Publishers, New York, 2nd edn, 1999) by using quinine sulfate in 0.10 M H_2_SO_4_ solution as the standard (Q = 0.54). The QY was calculated using the following Equation (1):


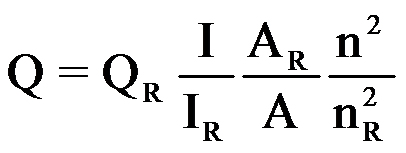
 (1)

Where *Q* is the quantum yield, *I* is the measured integrated emission intensity, *A* is the optical density, *n* is the refractive index (taken here as the refractive index of the respective solvents), and the subscript *R* stands for the reference fluorophore of known QY. We used UV-vis absorption spectrometer to determine the absorbance of the GQDs samples at 347 nm. The concentration of the GQDs for QY estimation should allow the first excitonic absorption peak to be below 0.05 in order to avoid any significant reabsorption. A Varian Cary Eclipse spectrophotometer was used to excite the samples at 347 nm and to record their photoluminescence spectra.

*Fluorescence lifetime*: The fluorescence lifetime (τ) of GQDs was determined by typical time-resolved photoluminescence measurement. As shown in Figure S21, the decay trace for GQDs is fitted by biexponential functions *Y(t)* based on nonlinear least-square as follows:


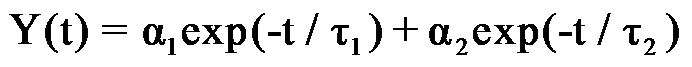
 (2)

Where α_1_ and α_2_ are the fractional contributions of time-resolved decay lifetime of τ_1_ and τ_2_, respectively. According to Equation (3):


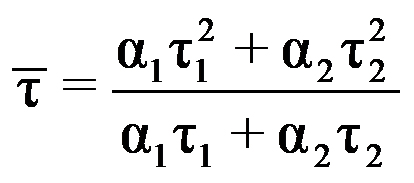
 (3)

We calculated the average lifetime ($\bar{\tau}$) of GQDs as 3.63 ± 0.05 ns (χ^2^< 1.1), which is comparable to the reported cases.^1, 2^

*Decomposition of Fe(VI) in reaction solution:* The characteristic color of ferrate solution is purple and this rapidly becomes to a yellowish color as decomposition occurs. The temporal change in Fe(VI) concentration can be observed by UV/vis spectroscopy. At a given wavelength (505-510 nm) and using a standard Fe(VI) solution with a range of concentrations, a calibration curve can be obtained and thus the Fe(VI) concentration can be measured. However, this direct spectrophotometric method has a limit that due to the high oxidation potential in aqueous solution Fe(VI) tends to rapidly decompose to Fe(III), which interfere with the optical monitoring of the solution. We applied an indirect spectrophotometric method to determine the concentration changes of Fe(VI)in the FIGO process by using the benign reagents: sodium iodide (NaI).^3, 4^ In this method, the reaction solution in the first stage of FIGO was taken out every 15 mins to mix with a large excess of I^-^. The purple color of mixture disappeared instantaneously and a yellow colored solution appeared. The reaction process is summarised in Equation (4):


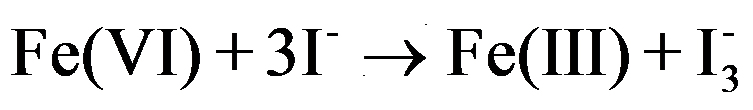
 (4)

Figure S3 demonstrates the spectra obtained for the mixed solutions, which had absorbance peaks at 287 nm and 349 nm. The characteristics of the spectra at 349 nm are ascribed to those of I_3_^−^ species and the peak at 287 nm may result from absorbance of the residual I^−^ and the Fe(III) product. The decreases in absorbance at 287 nm and 349 nm were linear with respect to the concentration of Fe(VI). The experiments were conducted for 120 mins, in which the raw graphite was oxidized as the reduction of Fe(VI) to Fe(III).

**Results**

*FIGO of GO Fabrication:* Graphite is known as a lamellar and planar structure. In single 2D layer, the carbon atoms in graphite are arranged in a honeycomb-like lattice and each layer is held together with the aid of weak van der Waals. There is large number of unsaturated aliphatic double bonds in these π-structures, which might serve as seed points for “unzipping” oxidation process.^5^ Therefore, we employ Fe(VI) to selectively oxidize the electron-rich moieties of localized defects in graphite sheet.^6^ Fe(VI) ion has the formula FeO_4_^2-^, and the reductive pathway of Fe(VI) to Fe(III) can be represented as equation (5). During this stage, the characteristic color change of black-purple (Fe(VI)) to brown color (Fe(III)) can be visually observed. For accurate determination of the valence states of Fe, we applied an indirect spectrophotometric method to examine the variation of FeO_4_^2-^ concentration every 15 mins (Figure S2). In the spectra, two peaks at 288 nm and 351 nm were observed. The absorbance at 288 and 351 nm decrease as time pass and the reduction rate rises more and more rapidly because of the accelerating decay of Fe(VI) in the presence of Fe(III). After 120 mins, the peaks doesn’t change any more, which can be ascribed to the complete conversion of Fe(VI) to Fe(III). As a result, raw graphite was partially oxidized by Fe(VI) (Figure S3-S6). Oxygen-containing groups might randomly spread across the defects or edge-atom sites in the graphitic lattice, giving rise to an intercalated and expanded structure.


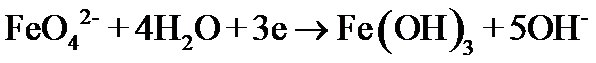
 (5)


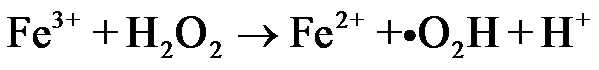
 (6)


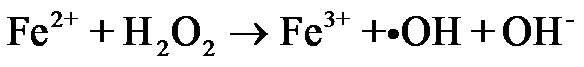
 (7)

In the second stage, the remaining Fe(III) derived from the Fe(VI) treatment works as a catalyst, H_2_O_2_ as a radical producer, and ultrasound as an accelerator and separator, respectively.^7^ The Sono-Fenton reaction forms active oxygen species as equations (6, 7). This process allows Fe(III) reproduction in an effective cycle that the generated Fe(II) can be oxidized by reaction with H_2_O_2_ to produce Fe(III) again and more radicals. On the basis of partially oxidized graphite via Fe(VI) oxygenation, the Fenton solution has an easy access to break into the interlayer and decorate both side of the flake sheet. Meanwhile, the introduction of ultrasound wave creates acoustic cavitation, resulting in the expansion and compression cycle of gas and vapor. Thousands of cavitation bubbles grow and impose in this process that facilitates the exfoliation of graphite oxide to GO. Together with the purification process, the whole process of FIGO only takes about 6 hours and residues are easily separated to Fe(III)/Fe(II) aqueous solution and unoxidized graphite, which can be recycled to use as the raw material in next FIGO synthesis, respectively.

*Partially oxidized graphite：*The preparation of partially oxidized graphite is accomplished by the oxidation reaction of raw graphite with Fe(VI) in **Stage-1** of FIGO (see Methods), in which Fe(VI) serves as an effective oxidant. The characterizations of partially oxidized graphite are shown in Figure S3-S6. Firstly, Raman measurement of the resultant sample was conducted. The G peak at 1580 cm^-1^ and D peak at 1360 cm^-1^ are observed, which are different from those of raw graphite and GO (Figure 2D). The presence of defects leads to the appearance of D peaks, while the G peak is assigned to double-degenerate *E*_2g_.^8^ It should be noted that the higher the value of D/G intensity ratio, the higher the lattice distortion of sample. As compared with graphite and GO, The D/G intensity ratio of sample is lower than that of FIGO-prepared GO and higher than raw graphite. These results imply that the sp^2^ fraction of graphite was interrupted by a certain number of oxygen functionalities in the **Stage-1** of FIGO, resulting in the loss of aromatic domain and the increase of defective structure. However, Fe(VI) does not seem able to oxidize the graphite thoroughly. After the **Stage-2** of FIGO, it can be observed in Figure 2D that D/G increases as well as the number of defects, indicating the higher oxidation degree.

Additionally, the XPS measurement recorded for the sample also support the conclusion that graphite is partially oxidized by Fe(VI) in the **Stage-1** of FIGO. The elemental composition of sample shows C/O = 8.0 in Figure S6. The C 1s XPS spectra display signals at ~284.5, ~286.6 and ~288.0 eV, which correspond to the carbons of aromatic sp^2^ regions (C-C/C=C), the carbons of hydroxyl and epoxy groups (C-OH/C-O-C) and carbons from carboxyl groups (C=O/O-C=O), respectively. It confirms that Fe(VI) aqueous solution produces sufficient oxidation species to break the C-C bonds and form sp^3^ defects. These defects with dangling group provide some active sites, which offer more opportunities for intercalation and exfoliation of Sono-Fenton reaction in the **Stage-2** of FIGO.

*Graphene Quantum Dots (GQDs):* GQDs were readily fabricated by using GO as the starting materials via a simple pyrolysis process.The resultant aqueous solution of GQDs displays interesting optical properties. Compared to GO solution, the solution of GQDs presents colorless under visual light and bright blue fluorescence under UV light (λ_ex_ = 365 nm). The emission spectra of sample exhibit typical excitation-dependent behaviour that the photoluminescence (PL) peak moves to longer wavelength as well as the enhancement of the excitation wavelength (Figure S17). When excited at 360 nm, the GQDs show strong PL emissions locating at 440 nm, in agreement with that of previously reported GQDs.^9, 10^ Figure S20 shows obvious absorption feature at ~ 270 and ~335 nm in their UV-vis absorption spectra. And the QY of GQDs were determined to reach 11.74 %. Moreover, time-resolved PL measurements demonstrate that GODs originated from FIGO-prepared GO have decay lifetimes of 3.63 ± 0.05 ns (Figure S19), using quinine sulfate as reference. It is of value for their applications, for instance, in optoelectronic device, sensor and optical imaging.


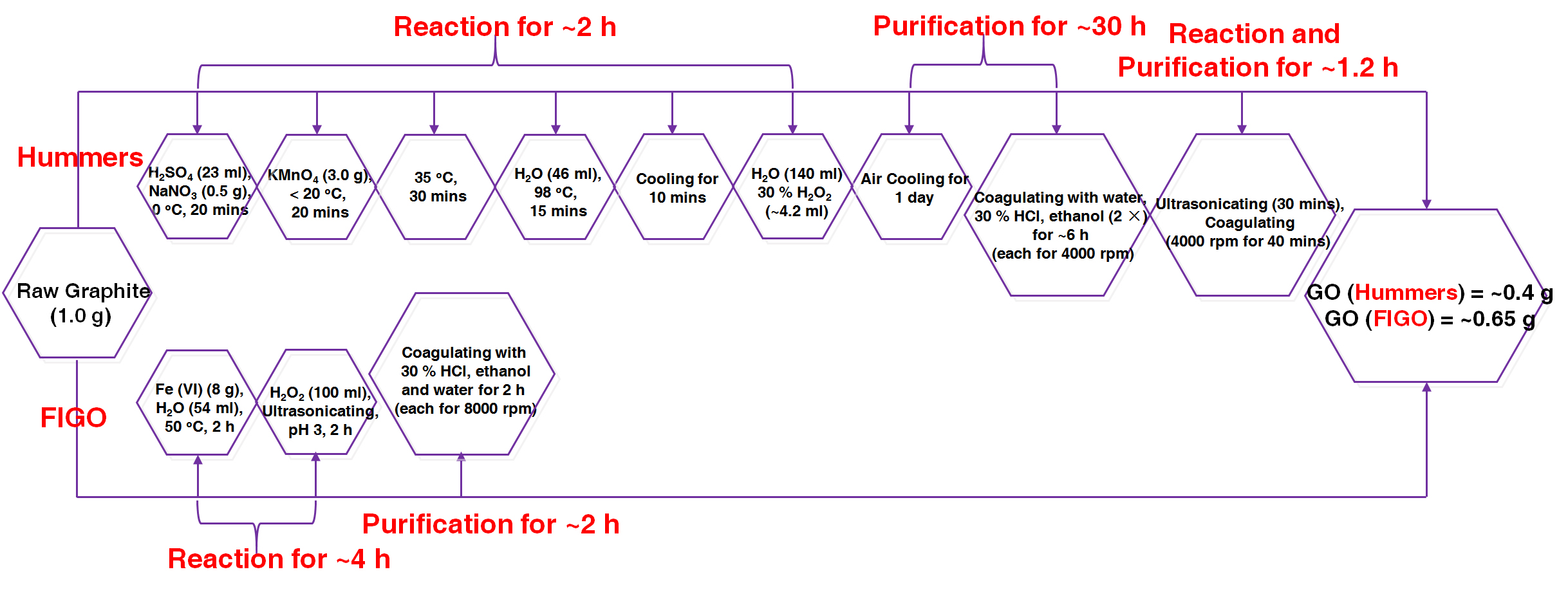


Figure S1. Comparison between Hummers’ method and FIGO for preparation of GO.

Numerous efforts have been made in the reduction of GO to graphene such as thermal exfoliation, chemical treatment and multi-step reduction*.*^11-14^ However, there are few developments in the oxidation of graphite to GO. The oxidation of graphite was first described by Schafhaeutl in 1840 and he referred to the product as “graphitic acid”.^15^ In 1859, Brondie used potassium chlorate (KClO_3_) as oxidant to oxidize the graphite in nitric acid and he discovered the synthesis of yellow graphitic acid.^16^ In 1898, Staudenmaier optimized this protocol by adding the chlorate in multiple aliquots over the reaction course to minimize the risk of explosions and using concentrated sulfuric acid (H_2_SO_4_) to increase the acidity of the mixture.^17^ Nearly 10 years later, Charpy first developed using the potassium permanganate (KMnO_4_) as the oxidant to oxidize the graphite in H_2_SO_4_ below 45 ^o^C.^18^ The same procedure was then optimized and scaled up in 1958, which is the most commonly used method today: Hummers’ method. Hummers treated the raw graphite with KMnO_4_ and NaNO_3_ in concentrated H_2_SO_4_ and applied this procedure on a multigram scale.^19^ The obtained produce called graphite oxide can be delaminated to single layers in suitable solvent, which was then named GO. Though many modified versions have been proposed in the following 56 years,^20-22^ these three procedures of Brondie, Staudenmaier and Hummers comprise the primary routes for the synthesis of GO, and little about them has changed. In the Figure S1, we compares Hummers’ method with our new FIGO in preparation of GO. The simpler operation, higher yield and non-toxic by-products of FIGO as compared to Hummers’ method are apparent.


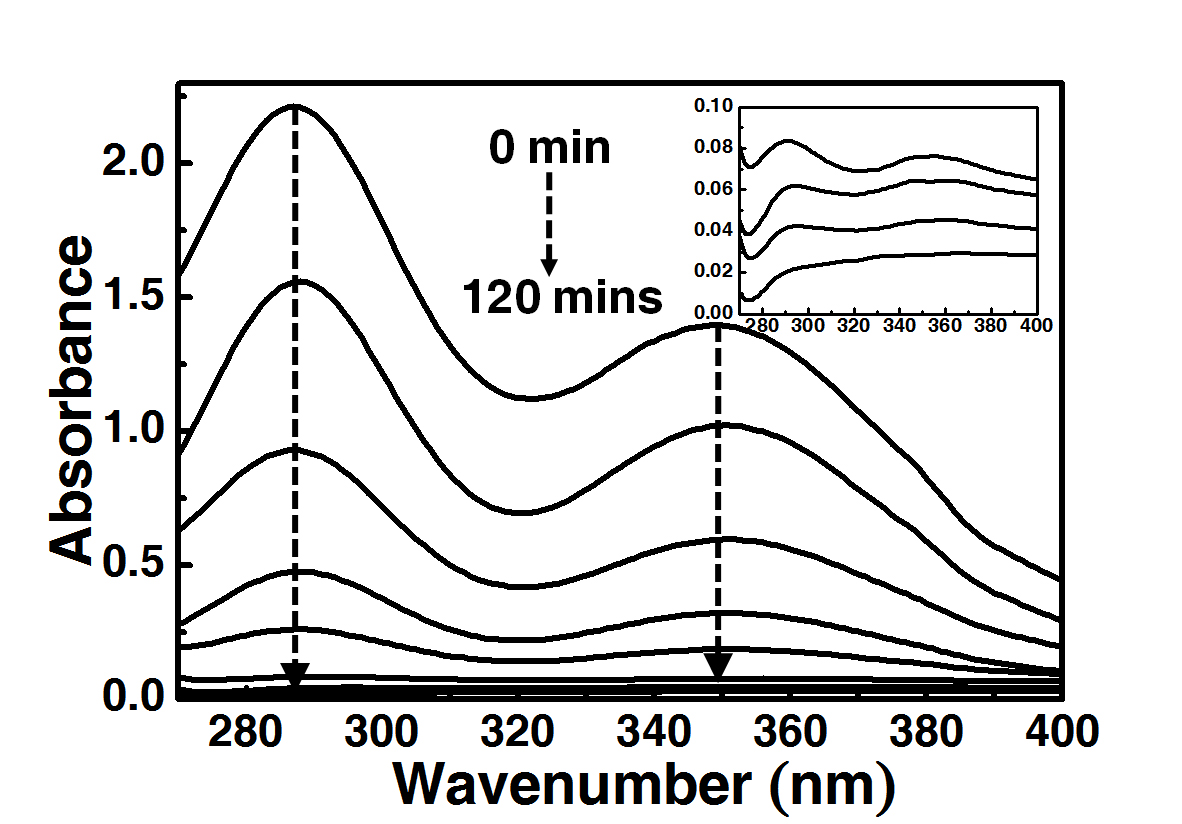


Figure S2. UV/vis absorption spectra of Fe(VI) in reaction solution as a function of time (0~120 mins). The inset diagram shows the UV/vis spectra from 75 to 120 mins. The obvious absorbance peaks at 287 nm and 549 nm are related to the variation of Fe(VI) concentration. The decreases of peaks indicate the decrease in the concentration of Fe(VI). As the reaction runs, the reduction rate of Fe(VI) accelerates because the generation of Fe(III) can catalyze the reaction of Fe(VI) and graphite. After 120 mins, the peaks don’t change any more, indicating the complete reaction of the first stage of FIGO.


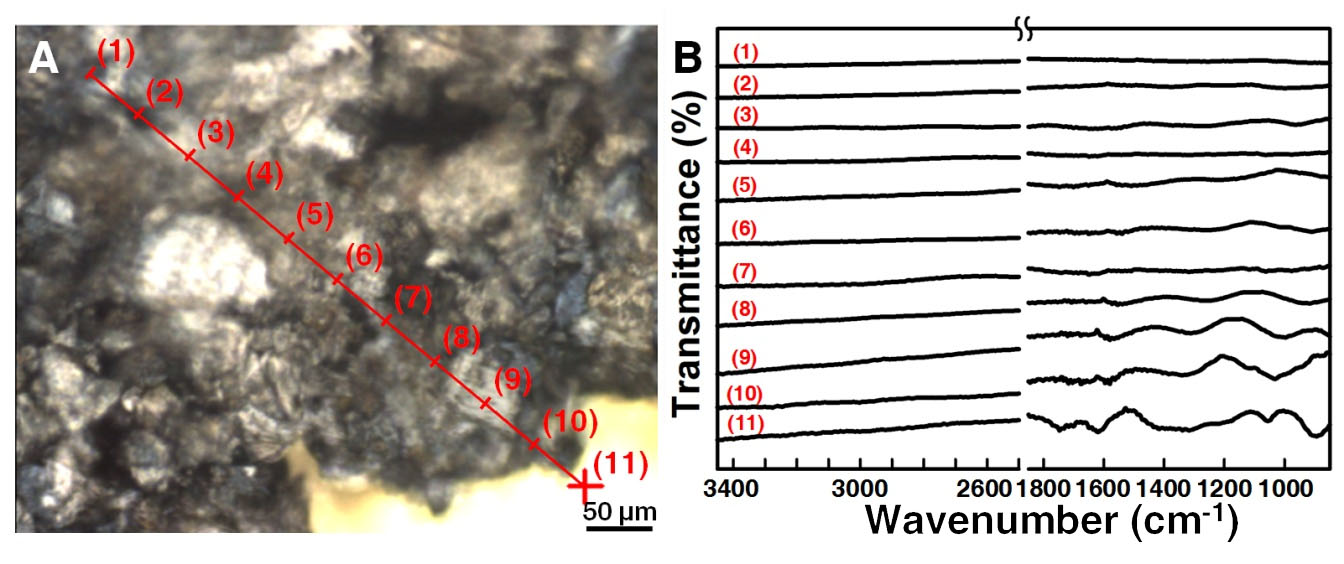


Figure S3. Optical microphotographs of partially oxidized graphite after the **Stage-1** of FIGO (A) and the corresponding IR spectra from certain areas (B). The IR spectra obtained from different spots (1-11) are very different. As is evident in right figure, the characteristic peaks of (11) are significantly observed compared to those of (1). There are almost no distinctive peaks in the spectra of (1)-(4), which are similar to that of the raw graphite. The peaks of C=O and C-O (1650~1580 cm^-1^ and 1150~1100 cm^-1^, respectively) appear as the detecting point moves from (5) to (8), suggesting the decoration of few functional groups. Furthermore, the intensities of oxygen-containing functional groups strongly enhance in the edge areas from (9) to (11). It might be explained that the **Stage-1** of FIGO is mainly performed on the periphery areas of raw graphite. Fe(VI) treatment unzips the graphitic layers, leading the formation of partially oxidized graphite.


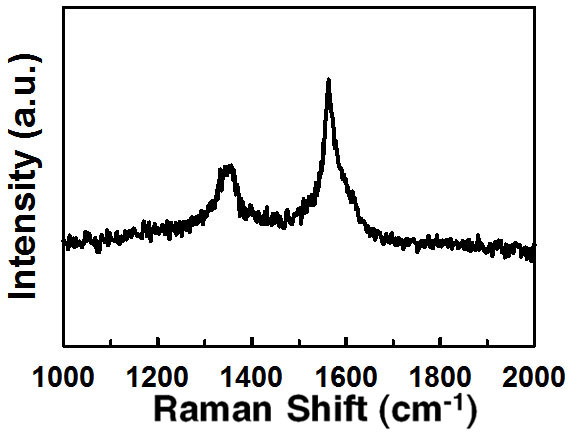


Figure S4. Raman spectrum of partially oxidized graphite obtained in **Stage-1**. The Raman spectrum demonstrates the structural changes occurring in sample. The G peak at 1580 cm^-1^ is due to the high-frequency *E*_2g_ phonon at **Γ** and the D peak at 1360 cm^-1^ corresponds to the *A*_1g_ breathing modes at **K**. The sp^3^ hybridization caused by Fe(VI) generates the D peak, accompanying with the damages of sp^2^ carbon framework. In the **Stage-1** of FIGO, Fe(VI) introduces a range of oxygen-containing groups to break the weak van der Waals force of raw graphite.


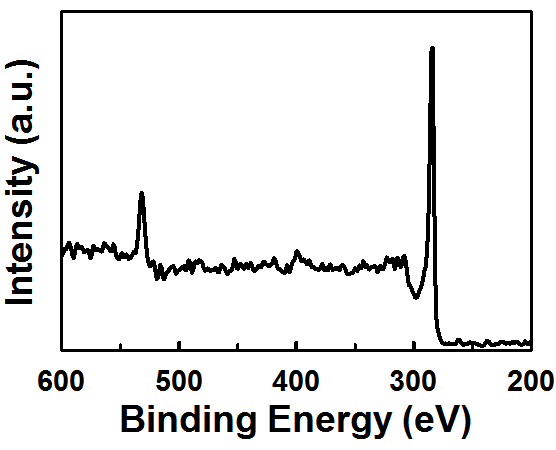


Figure S5. XPS curve of partially oxidized graphite obtained in **Stage-1**. The elemental composition of sample reveals that C/O atomic ratio (C/O = 8.0) of sample is lower than that of FIGO-obtained GO (Figure S14), which confirm the partial oxidation of graphite in the **Stage-1** of FIGO.


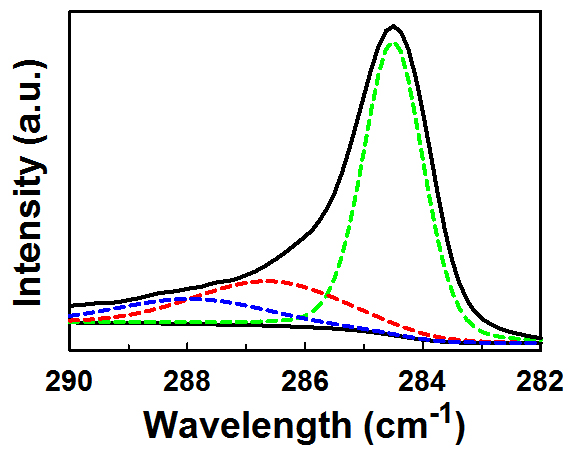


Figure S6. C 1s curve of partially oxidized graphite obtained in **Stage-1**. The XPS spectrum shows three peaks at ~284.5, ~286.6 and ~288.0 eV, which belong to non-oxygenated aromatic carbon, C–O and C=O bonds, respectively. It should be mentioned that the peaks for C–O and C=O groups are weaker than those of FIGO-obtained GO (Figure 2F), indicating the low level of oxidation in the **Stage-1**.


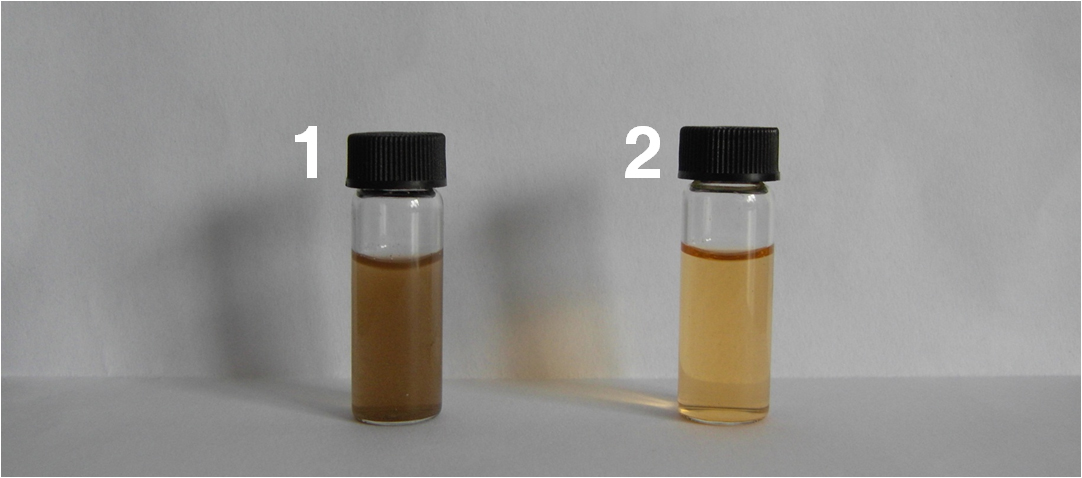


Figure S7. Photographs of the as-prepared GO without purification (sample 1) and after purification (sample 2). The difference can be visually distinguished that sample 1 shows brown while sample 2 is light yellow in color.


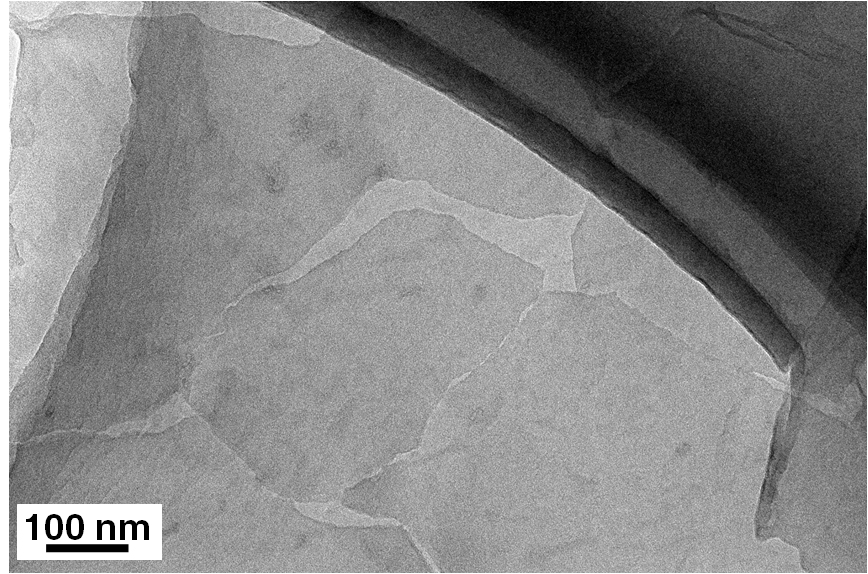


Figure S8. TEM image of sample 2. After purification, GO platelets were obtained.


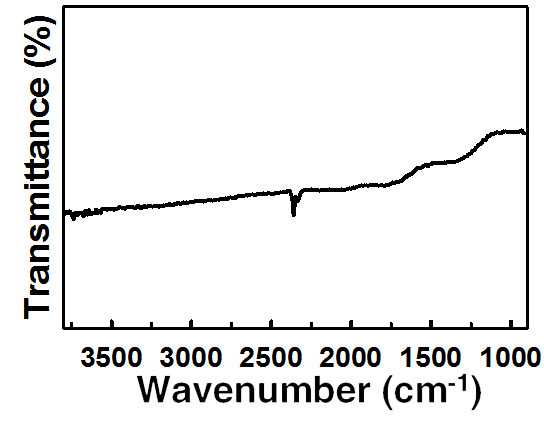


Figure S9. IR spectrum of graphite. There are no distinctive peaks in raw material.


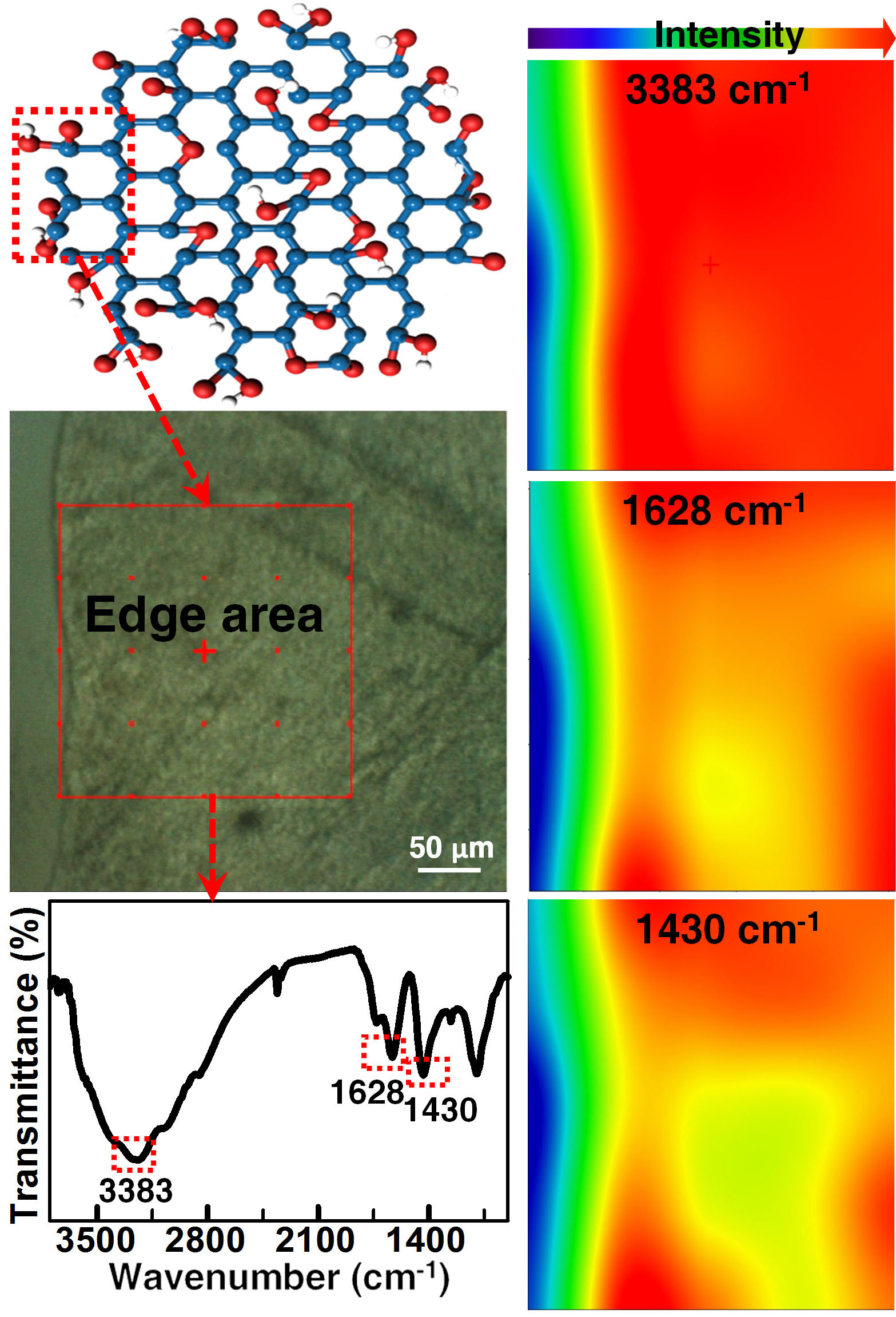


Figure S10. Optical (left) and IR (right) images of the edge area of a GO flake prepared via FIGO. As evidenced in the IR spectrum, O-H stretching vibrations for carboxyl and hydroxyl groups locate at 3383 cm^-1^, unoxidized C=C group centers at 1628 cm^-1^, and the O-H bending vibration appears at 1430 cm^-1^. The relative absorption intensities of groups are indicated by the color changes that red color means high intensity and blue color represents low intensity.


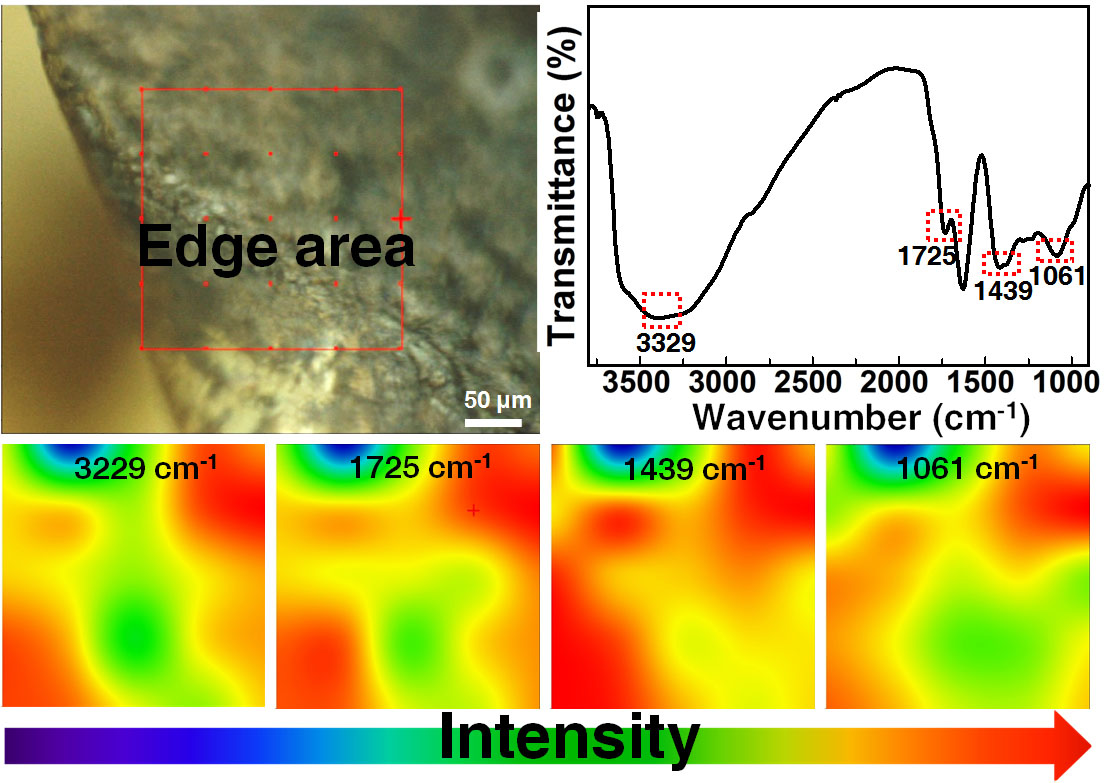


Figure S11. Optical (top) and IR (down) images of the edge area of another GO flake sample prepared via FIGO. The IR spectrum demonstrates that O-H stretching vibrations for carboxyl and hydroxyl groups center at 3229 cm^-1^, C=O group locates at 1725 cm^-1^, the O-H bending vibration appears at 1439 cm^-1^, and the C-O epoxy group is related to the band at 1061 cm^-1^. The relative absorption intensities of groups are indicated by the color changes that red color means high intensity and blue color represents low intensity.


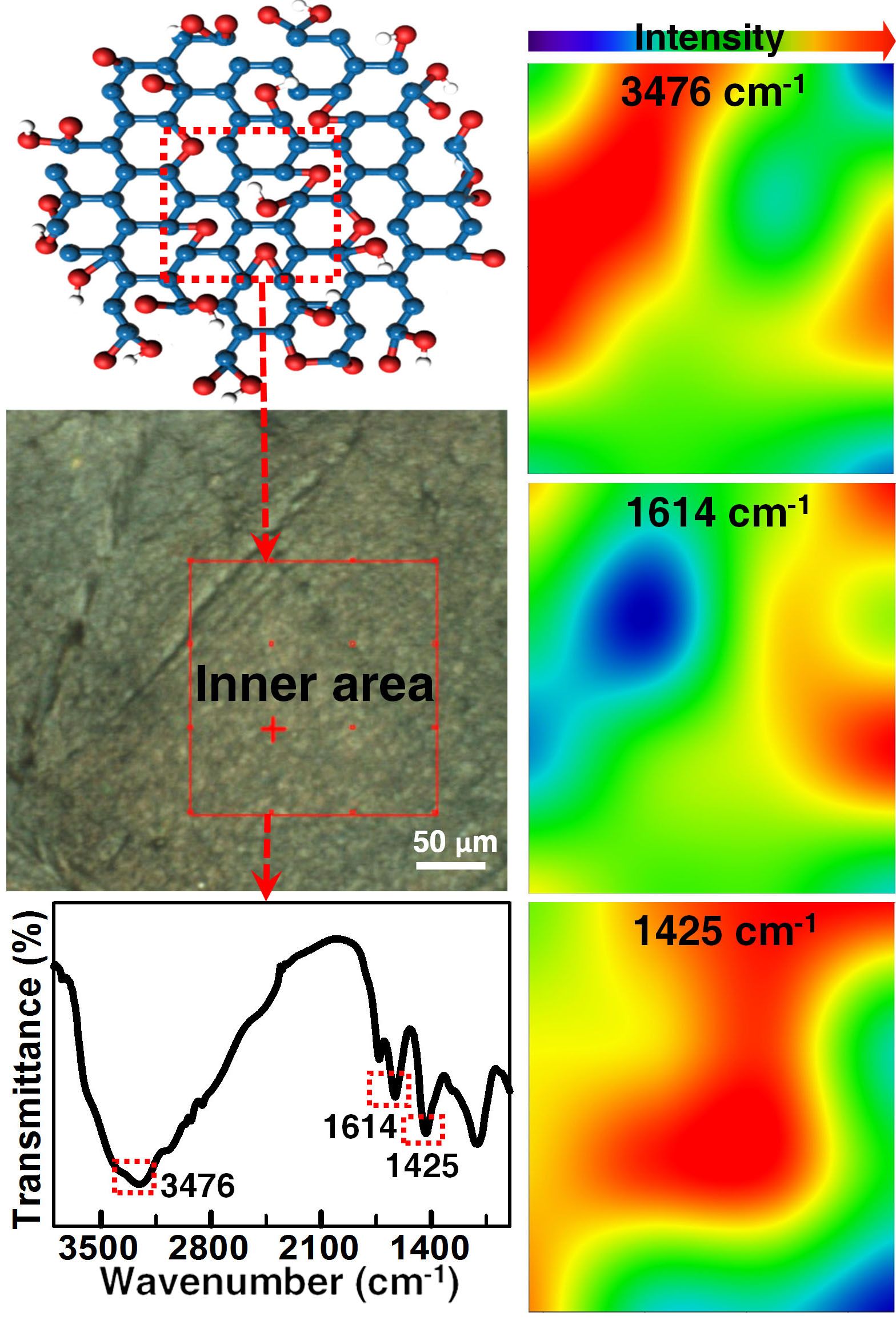


Figure S12. Optical (top) and IR (down) images of the inner area of a GO flake prepared via FIGO. As shown in the IR spectrum, O-H stretching vibrations for carboxyl and hydroxyl groups center at 3476 cm^-1^, unoxidized C=C group locates at 1614 cm^-1^, and the O-H bending vibration appears at 1425 cm^-1^. The relative absorption intensities of groups are indicated by the color changes that red color means high intensity and blue color represents low intensity.


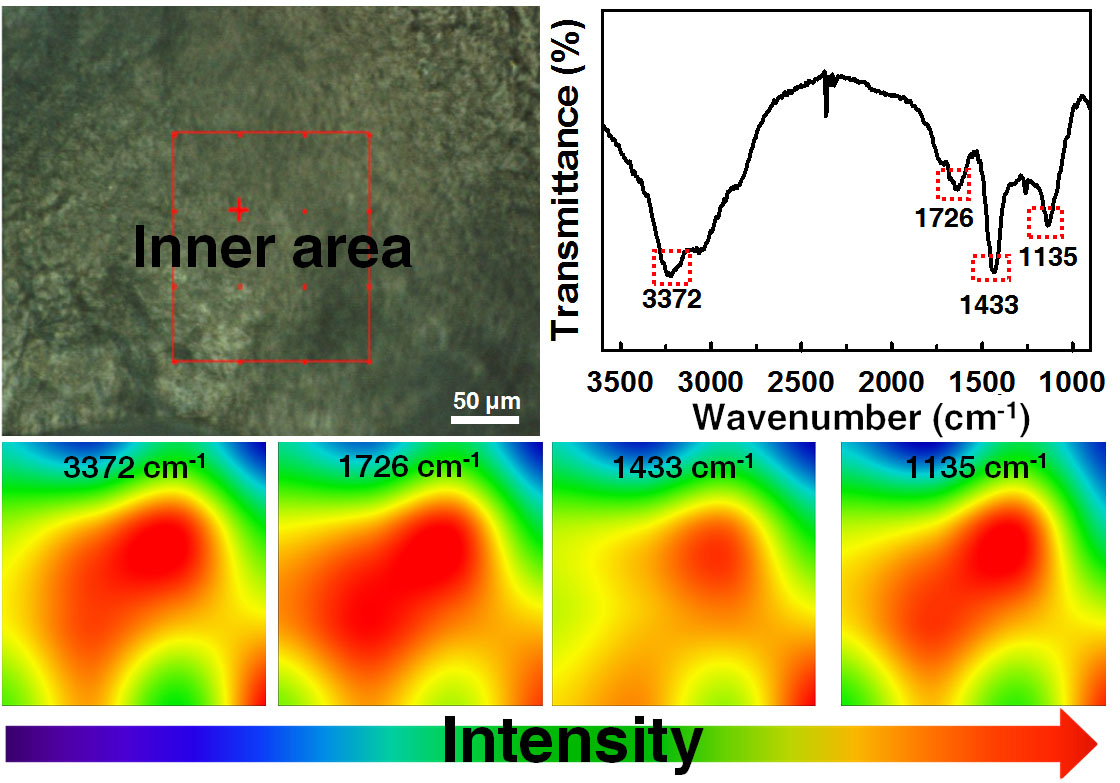


Figure S13. Optical (top) and IR (down) images of the inner area of another GO flake sample prepared via FIGO. The IR spectrum demonstrates that O-H stretching vibrations for carboxyl and hydroxyl groups center at 3372 cm^-1^, C=O group locates at 1726 cm^-1^, the O-H bending vibration appears at 1433 cm^-1^, and the C-O epoxy group is related to the band at 1135 cm^-1^. The relative absorption intensities of groups are indicated by the color changes that red color means high intensity and blue color represents low intensity.


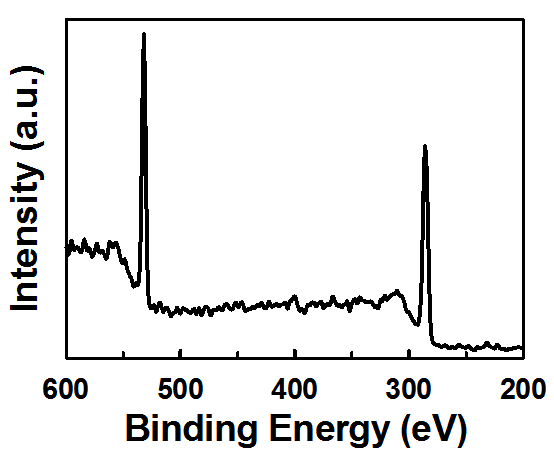


Figure S14. XPS curve of GO. The elemental composition of GO flake prepared via FIGO has C/O atomic ratio of 2.2, suggesting the presence of oxygen functionalities.


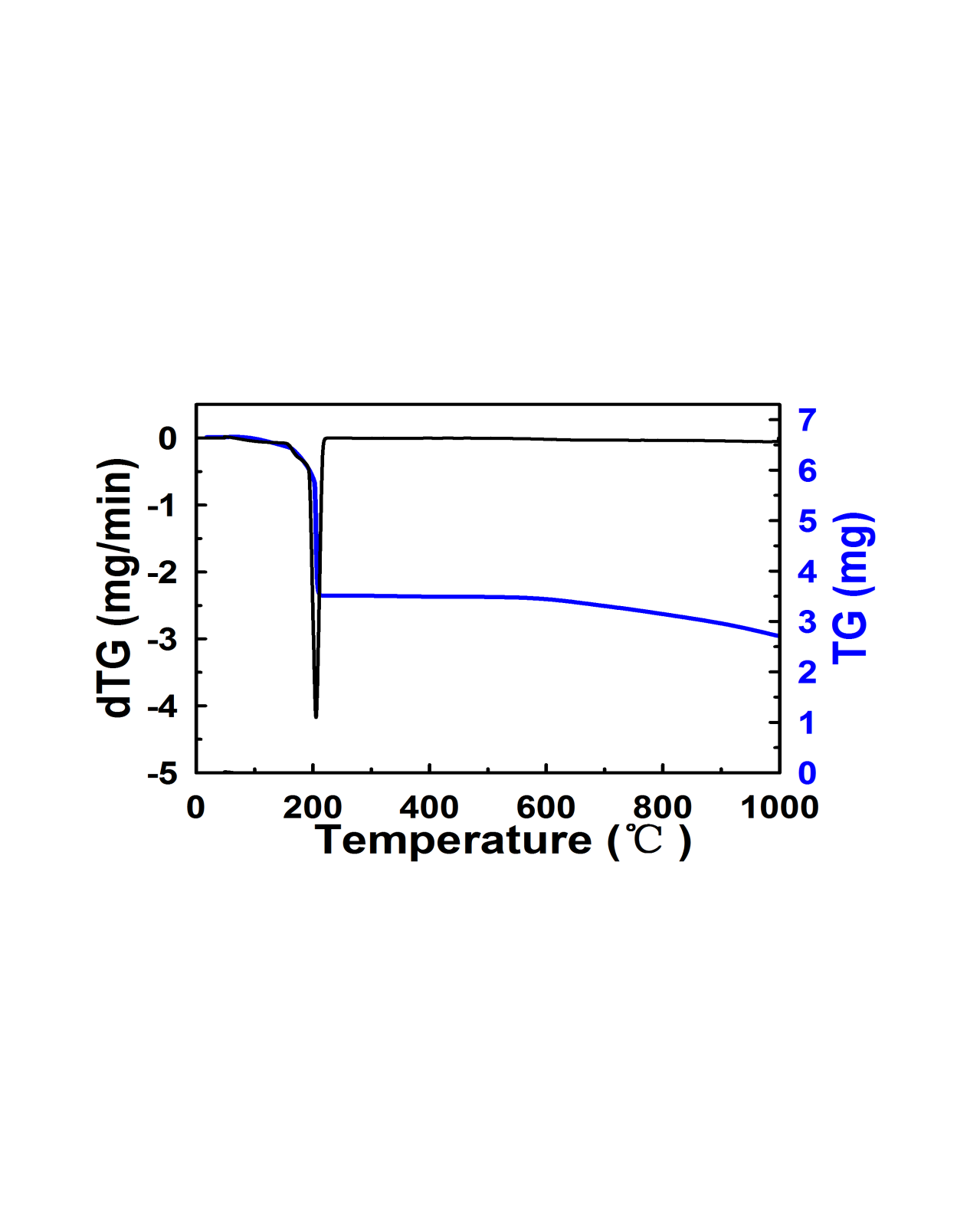


Figure S15. TG (blue) and DTG (black) curves of FIGO-obtained.


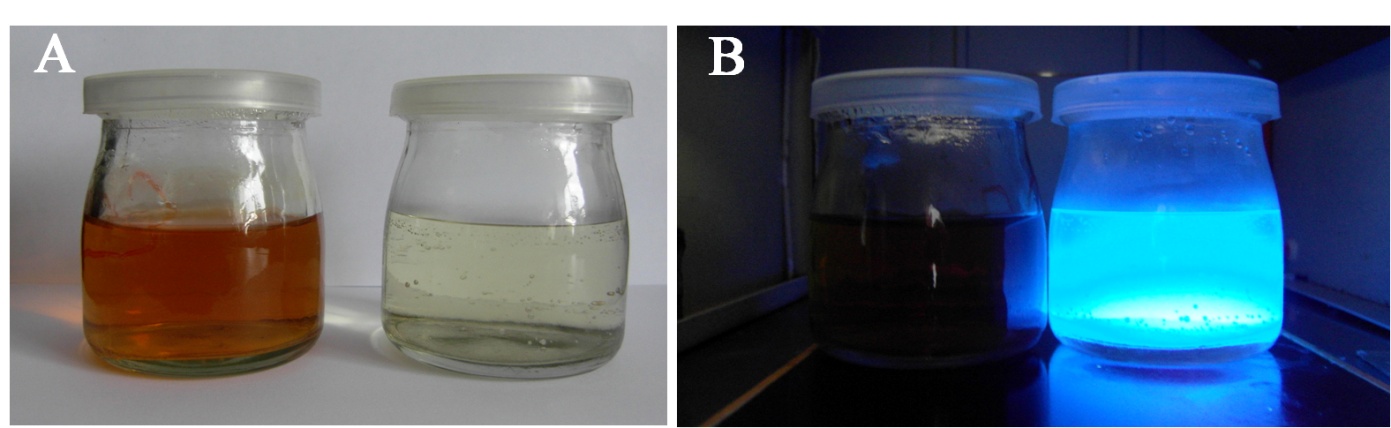


Figure S16. (A, B) Photographs of the aqueous solutions of FIGO-obtained GO and GQDs under daylight (A) and UV light (B, λ_ex_ = 365 nm).


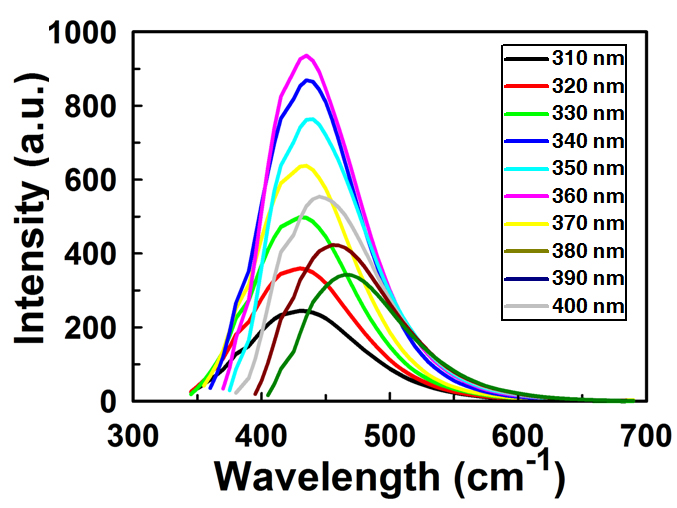


Figure S17. PL spectra of an aqueous solution of GQDs at different excitation wavelength (in 10 nm increment starting from 310 nm to 400 nm).


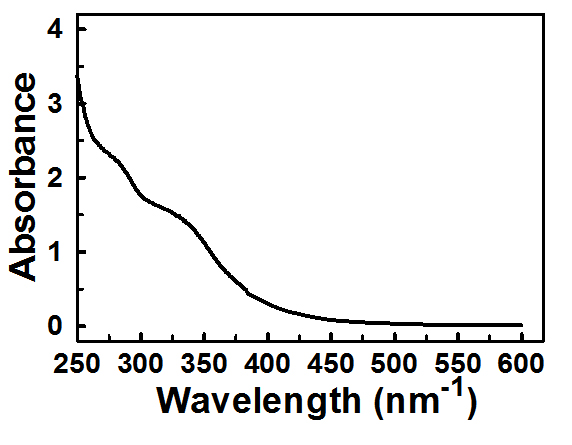


Figure S18. UV-vis absorption spectrum of an aqueous solution of GQDs.


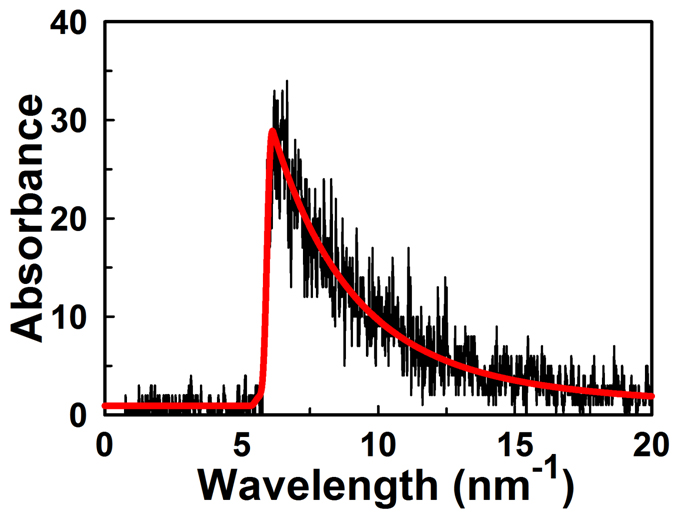


Figure S19. Time-resolved fluorescence decay curves of GQDs measured at 405 nm laser excitation.


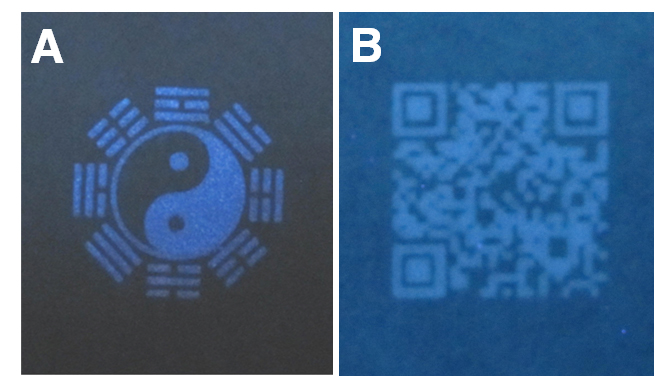


Figure S20. Photograph of the fluorescent pattern by screen printing (A) and inkjet printing (B) under UV light.


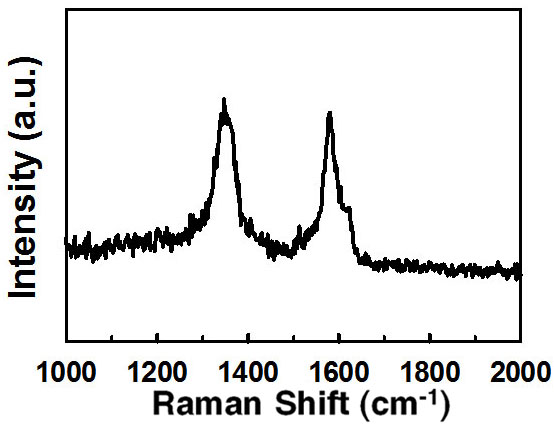


Figure S21. Raman spectrum of graphene prepared from FIGO-prepared GO. The graphene was prepared from the reduction of as-prepared GO with N_2_H_4_ and NH_3_ according to the literature method.^12^


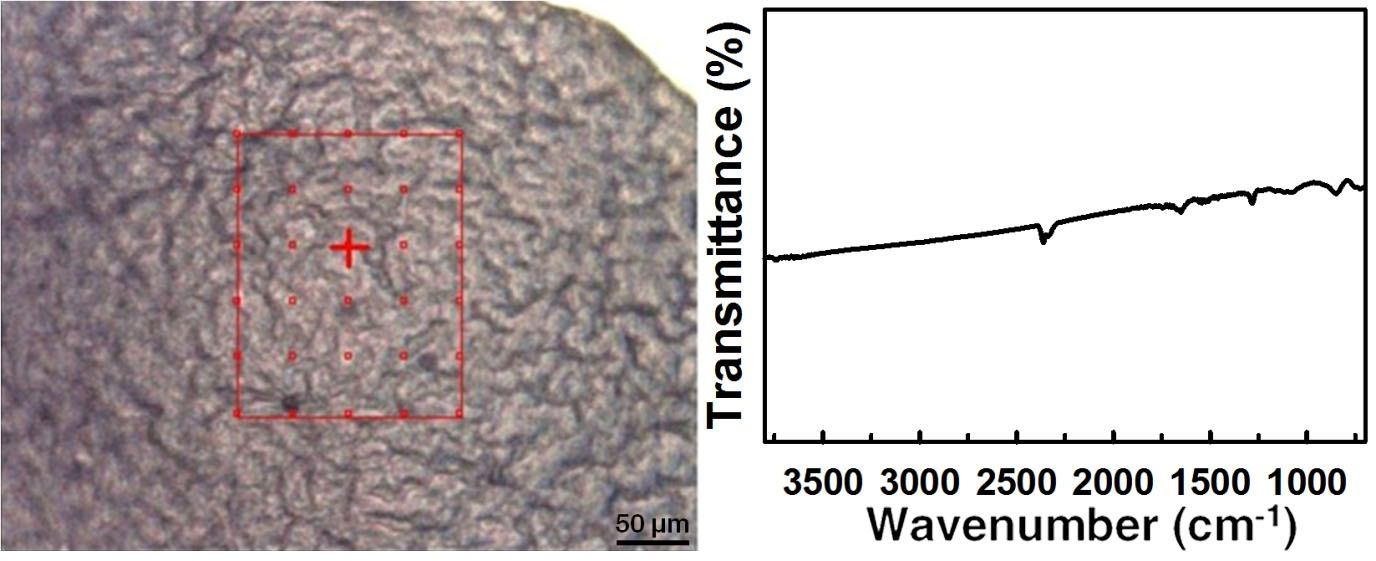


Figure S22. Optical image (left) and FTIR spectrum (right) of graphene prepared from FIGO-prepared GO. The graphene was prepared from the reduction of as-prepared GO with N_2_H_4_ and NH_3_ according to the literature method.^12^


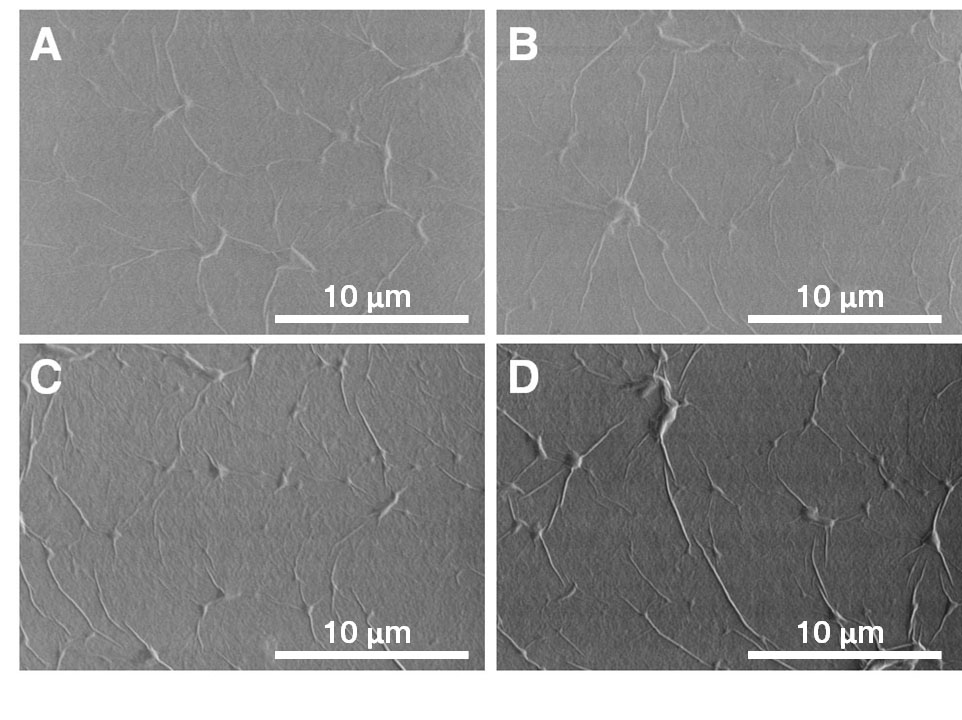


Figure S23. SEM micrograph of FIGO-prepared GO.

Table S1. XPS determined compositions of samples from **Stage-1** and **Stage-2**.

| XPS Atomic Concentrations (%) | | | | |
| --- | --- | --- | --- | --- |
|  | C | O | Fe | S |
| Sample from **Stage-1** | 82.14 | 10.47 | 7.39 | 0.00 |
| Sample from **Stage-2** | 65.45 | 29.89 | 4.66 | 0.00 |

This XPS data corresponds to the data shown in Figure 2, S5 and S6. Significantly, large content of oxo-speices is introduced upon FIGO. For both samples, there is no S observed.

Reference

(1) Zhong, H. Z.; Zhou, Y.; Ye, M. F.; He, Y. J.; Ye, J. P.; He, C.; Yang, C. H.; Li, Y. F. Controlled Synthesis and Optical Properties of Colloidal Ternary Chalcogenide CuInS2 Nanocrystals. *Chem. Mater.* **2008**, *20*, 6434-6443.

(2) Yang, S. Y.; Wang, C. F.; Chen, S. Interface-Directed Assembly of One-Dimensional Ordered Architecture from Quantum Dots Guest and Polymer Host. *J. Am. Chem. Soc.* **2011**, *133*, 8412-8415.

(3) Klassen, N. V.; Marchington, D.; Mcgowan, H. C. E. H2o2 Determination by the I-3(-) Method and by Kmno4 Titration. *Anal. Chem.* **1994**, *66*, 2921-2925.

(4) Luo, Z. Y.; Strouse, M.; Jiang, J. Q.; Sharma, V. K. Methodologies for the analytical determination of ferrate(VI): A Review. *J. Environ. Sci. Heal. A* **2011**, *46*, 453-460.

(5) Zhu, Y. W.; Murali, S.; Cai, W. W.; Li, X. S.; Suk, J. W.; Potts, J. R.; Ruoff, R. S. Graphene and Graphene Oxide: Synthesis, Properties, and Applications (vol 22, pg 3906, 2010). *Adv. Mater.* **2010**, *22*, 5226-5226.

(6) Jiang, J. Q. Advances in the development and application of ferrate(VI) for water and wastewater treatment. *J. Chem. Technol. Biot.* **2014**, *89*, 165-177.

(7) Iordache, I.; Wilson, S.; Lundanes, E.; Iordache, M.; Pavel, V. L.; Aelenei, N. The Fenton and Sono-Fenton Processes Applied for Pesticide Degradation. *Environ. Eng. Manag. J.* **2010**, *9*, 519-525.

(8) Ferrari, A. C.; Basko, D. M. Raman spectroscopy as a versatile tool for studying the properties of graphene. *Nat. Nanotechnol.* **2013**, *8*, 235-246.

(9) Jaiswal, A.; Ghosh, S. S.; Chattopadhyay, A. One step synthesis of C-dots by microwave mediated caramelization of poly(ethylene glycol). *Chem. Commun.* **2012**, *48*, 407-409.

(10) Wang, J.; Wang, C. F.; Chen, S. Amphiphilic Egg-Derived Carbon Dots: Rapid Plasma Fabrication, Pyrolysis Process, and Multicolor Printing Patterns. *Angew. Chem. Int. Edit.* **2012**, *51*, 9297-9301.

(11) Eda, G.; Fanchini, G.; Chhowalla, M. Large-area ultrathin films of reduced graphene oxide as a transparent and flexible electronic material. *Nat. Nanotechnol.* **2008**, *3*, 270-274.

(12) Li, D.; Muller, M. B.; Gilje, S.; Kaner, R. B.; Wallace, G. G. Processable aqueous dispersions of graphene nanosheets. *Nat. Nanotechnol.* **2008**, *3*, 101-105.

(13) Chua, C. K.; Pumera, M. Chemical reduction of graphene oxide: a synthetic chemistry viewpoint. *Chem. Soc. Rev.* **2014**, *43*, 291-312.

(14) Kumar, P. V.; Bardhan, N. M.; Tongay, S.; Wu, J. Q.; Belcher, A. M.; Grossman, J. C. Scalable enhancement of graphene oxide properties by thermally driven phase transformation. *Nat. Chem.* **2014**, *6*, 151-158.

(15) Schafhaeutl, C. On the conbinations of carbon with silicon and iron, and other metals, forming the different species of cast iron, steel, and malleable iron. *Phil. Mag.* **1840**, *16*, 570-590.

(16) Brodie, B. C. On the Atomic Weight of Graphite. *Philos. Trans. R. Soc. London* **1859**, *149*, 249-259.

(17) Staudenmaier, L. Verfahren zur Darstellung der Graphitsa¨ ure. *Ber. Dtsch. Chem. Ges.* **1898**, *31*, 1481-1487.

(18) Charpy, G. Formation of Graphitic Oxide and the Definition of Graphite. *Compt. rend.* **1909**, *148*, 920-923.

(19) W. S. Hummers, R. E. O. Preparation of Graphitic Oxide. *J. Am. Chem. Soc.* **1958**, *80*, 1339.

(20) Marcano, D. C.; Kosynkin, D. V.; Berlin, J. M.; Sinitskii, A.; Sun, Z. Z.; Slesarev, A.; Alemany, L. B.; Lu, W.; Tour, J. M. Improved Synthesis of Graphene Oxide. *Acs Nano* **2010**, *4*, 4806-4814.

(21) Bao, C. L.; Song, L.; Xing, W. Y.; Yuan, B. H.; Wilkie, C. A.; Huang, J. L.; Guo, Y. Q.; Hu, Y. Preparation of graphene by pressurized oxidation and multiplex reduction and its polymer nanocomposites by masterbatch-based melt blending. *J. Mater. Chem.* **2012**, *22*, 6088-6096.

(22) Chiu, P. L.; Mastrogiovanni, D. D. T.; Wei, D. G.; Louis, C.; Jeong, M.; Yu, G.; Saad, P.; Flach, C. R.; Mendelsohn, R.; Garfunkel, E.; He, H. X. Microwave- and Nitronium Ion-Enabled Rapid and Direct Production of Highly Conductive Low-Oxygen Graphene. *J. Am. Chem. Soc.* **2012**, *134*, 5850-5856.
